# Electroacupuncture Improves the Survival Rate and Organ Function in a Rat Model of Hemorrhagic Shock

**DOI:** 10.1155/2019/8371862

**Published:** 2019-12-11

**Authors:** Yuxian Zhong, Guochen Xu, Yushou Wu, Huiping Zhang, Haibin Wang, Liqian Ma, Wenhua Zhang, Yongming Yao, Lu Wang, Sen Hu, Gerhard Litscher

**Affiliations:** ^1^Department of Rehabilitation Medicine, The Six Medical Center of PLA General Hospital, Beijing 100048, China; ^2^Outpatient Department, The Fourth Medical Center of PLA General Hospital, Beijing 100037, China; ^3^Burn Institute, The Fourth Medical Center of PLA General Hospital, Beijing 100037, China; ^4^Research Laboratory of Trauma Surgery, The Fourth Medical Center of PLA General Hospital, Beijing 100037, China; ^5^Laboratory Medicine, The Fourth Medical Center of PLA General Hospital, Beijing 100037, China; ^6^Research Unit of Biomedical Engineering in Anesthesia and Intensive Care Medicine, Research Unit for Complementary and Integrative Laser Medicine and TCM Research Center Graz, Medical University of Graz, Auenbruggerplatz 39, 8036 Graz, Austria

## Abstract

Electroacupuncture (EA) at ST36 can improve the survival rate in rats after hemorrhagic shock (HS). The current study investigated rats with 60% blood loss. 144 rats were divided into four groups: hemorrhage without fluid resuscitation (HS), EA after hemorrhage without fluid resuscitation (EA), hemorrhage with delayed resuscitation (DFR), and EA after hemorrhage with delayed resuscitation (EA + DFR). The survival rate and biological parameters 0, 3, 12, and 24 h after HS were investigated. The 24 h survival rate of EA + DFR was significantly higher than that of DFR. 12 h after hemorrhage, the level of mean arterial blood pressure of EA + DFR was significantly higher than that of DFR, and the levels of renal blood flow, intestinal mucosal blood flow, and hepatic blood flow of EA + DFR were also significantly higher than those of DFR. Three hours after hemorrhage, the levels of lactate, PaCO_2_, alanine aminotransferase, and creatinine of groups receiving EA were significantly lower than those of non-EA groups, and the levels of pH, PaO_2_, and diamine oxidase of groups receiving EA were significantly higher. EA at ST36 can improve the 24 h survival rate and produce the experimental antishock effects on tissue perfusion and organ protection from fatal HS.

## 1. Introduction

Death from hemorrhage represents a substantial global problem, with an estimated 1.9 million deaths per year worldwide [1]. It has been reported that hemorrhagic shock (HS) is the main cause of potentially preventable trauma deaths, and fluid resuscitation or restoration of the intravascular volume as quickly as possible is considered the most effective measure for treating HS and reducing the death rate [2]. However, according to battlefield statistics reports, nearly 90% of the deaths occur before the wounded can be evacuated to a medical facility, with HS accounting for approximately 50% of these deaths because conventional fluid resuscitation is very difficult to implement in a timely fashion in battlefield environments [3]. Even though the injured can be transported to medical institutions within a few hours, there is still a high death rate after delayed fluid resuscitation for ischemia reperfusion injury (IRI) and multiple organ dysfunction syndrome (MODS) [4]. Therefore, it is important to explore new treatment measures for increasing a victim's tolerance of tissue hypoxia and resistance to reperfusion injury, which would eventually improve the survival rate of patients with HS in austere environments such as battlefields or scenes of accidents or disasters, where intravenous fluid resuscitation may be lacking.

Acupuncture, as an ancient Chinese method of cure using only needles, has been applied for thousands of years and used for emergency treatment by Asian doctors. Acupuncture needles, either manipulated manually or stimulated using a low current and frequency, have been documented to be a neurophysiological basis for modulating the activity of peripheral and central neural pathways and decreasing the organic oxygen demand, and many acupoints have been proven by modern research to be effective in treating shock [5–8]. Previous studies have demonstrated that acupuncture at the ST36 acupoint (Zusanli) can greatly improve the survival rate [9], elevate the blood pressure [10], and provide multiorgan protection, particularly for the heart [11], liver [12], and intestine [13, 14] in the HS rat model with 45% hemorrhage.

However, on the basis of previous studies, the blood loss volume of an animal model with HS treated by ST36 is mostly lower than 45%, which may not meet the requirement of a 60% blood loss volume for the battlefield environment [15]. Thus, we replicated a clinically relevant rat model with 60% blood loss and investigated whether EA at ST36 combined with delayed intravenous fluid resuscitation can protect against organ injury, thus increasing the survival rate for fatal HS.

## 2. Materials and Methods

### 2.1. Animals and Ethics Approval

Sprague-Dawley (SD) male (specific pathogen free) adult rats (weight 220 ± 7.8 g) were acclimatized under constant temperature and humidity for 7 days. They were deprived of food for 12 h and were kept from drinking for 4 h before surgical manipulations. This study was performed in accordance with the Guide for Care and Use of Laboratory Animals of National Research Council and approved by the Fourth Medical Center of PLA General Hospital, Beijing, China.

### 2.2. Surgical Procedures

Rats were anesthetized and instrumented with 3% isoflurane inhalation (Yeeran Technology Limited, Beijing, China), and anesthesia was maintained with isoflurane (0.7%) during the procedures. The rats were allowed to breathe spontaneously under a nose cone scavenging system using a veterinary anesthesia delivery system (Kent Scientific TOPO, Torrington, CT, United States). After the abovementioned operation, in sterile conditions, three poly-ethylene (PE50) catheters were placed in the right carotid artery for the measurement of the blood pressure, in the left femoral artery for drawing blood and in the left femoral vein for delayed fluid infusion. Then, the abdominal cavity was cut open along the ventral midline at approximately 4 cm to place the laser Doppler probes from the Laser Doppler flowmetry system (PeriFlux5000, PERIMED, Sweden). A 1% heparin saline solution was injected into the femoral vein to make the animals systemically heparinized. Next, the blood pressure and visceral blood flow were taken at baseline, which means 0.5 h before blood loss (T-0.5).

### 2.3. HS Protocol

We used the following formula [16] to calculate the systemic estimated blood volume of each rat: total blood volume (TBV) = weight (g) × 0.06 (mL/g) + 0.77. According to the improved method described in the previous study of Gonzales et al. [17], the rats were bled 60% of their TBV in two stages: to simulate femoral arterial blood loss and subsequent continuous venous bleeding in battlefield or accident environments, first, 40% of the TBV was withdrawn from the left femoral artery in 10 min, and then 20% of the TBV was withdrawn from the left femoral vein in 170 min. Bleeding was performed using withdrawal pumps (Kelifeng Apparatus, Beijing, China). Anesthesia was maintained at a level of 0.7% isoflurane in this process.

### 2.4. Experimental Protocols

**Experiment 1 .**

**Figure 1:**
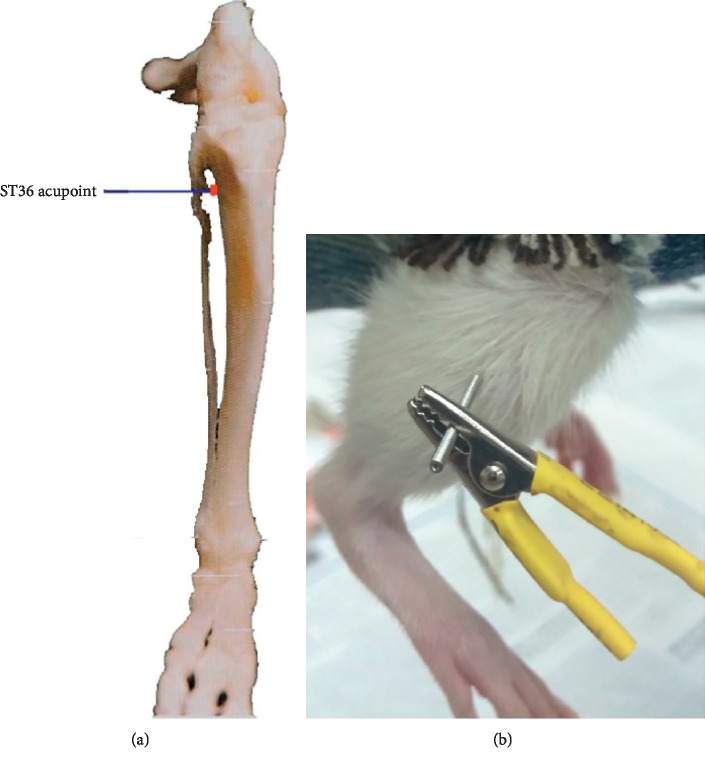
(a, b) The location of the ST36 acupoint on the SD rats [18].

Investigation of the Survival Rates, MAP, and Visceral Blood Flow. All rats underwent the same surgical procedure and HS protocol and were divided into the following four groups (18 animals each):

- Hemorrhagic shock group (HS): animals were subjected to 60% blood loss without intravenous fluid.
- Electroacupuncture group (EA): 0.5 h after arterial blood loss, animals were given EA at the ST36 acupoint bilaterally for 25 minutes, a frequency of 4 Hz, and a constant voltage of 4 V. The ST36 acupoint is located on the posterolateral side of the leg, 5 mm below the fibular head (see Figure 1). Two sterilized acupuncture needles (diameter 0.16 mm and length 25 mm) were inserted at the ST36 acupoint to a depth of 4 ± 5 mm, and the needles were connected by their handles to an electrostimulator (KWD-808I, YING DI, China) [18]. Animals in this group were not resuscitated with intravenous fluid.
- Delayed fluid resuscitation group (DFR): animals were intravenously resuscitated with sodium lactate Ringer's solution equal to 3 times the volume of blood loss at 3 h after hemorrhage and were not subjected to EA.
- Electroacupuncture with delayed fluid resuscitation group (EA + DFR): HS animals underwent EA and delayed intravenous fluid. The EA method was the same as the EA group. The infusion method was the same as the DFR group.

The survival rates of each group were recorded at 0 h (T0), 3 h (T3), 12 h (T12), and 24 h (T24) after the HS protocol. The MAP and visceral blood flow were measured at T-0.5, T0, T3, and T12 using a cardiorespiratory monitor (Power Lab, AD Instruments Pty Ltd, Australia) and a laser Doppler flowmetry system (PeriFlux5000, PERIMED, Sweden).

**Experiment 2 .**

Investigation on Blood Gas and Organ Function. Another 72 rats underwent the same protocols and were then divided into four groups as in experiment 1. At T3, all surviving animals were euthanized with bleeding of the abdominal aorta. The jejunum, kidney, and liver were harvested and assessed for the tissue water content using the wet-dry weight method [19], and diamine oxidase (DAO) from the jejunum was measured with reagents (DAO kits, Jian Cheng Technology Ltd, China). Arterial blood samples were drawn and measured for pH, PaCO_2_, PaO_2_, and lactate (LA) with a blood gas analyzer (Roche, Mannheim, Germany). Plasma parameters of organ function, such as alanine aminotransferase (ALT) and creatinine (Cr), were determined using a VT-5600 analyzer (Johnson & Johnson, New Jersey, USA).

### 2.5. Statistical Evaluation

Statistical analyses of data were performed with the SPSS for Windows v.10.0 software program. Continuous variables were expressed as the mean ± standard deviation. The statistical significance of the differences between the groups was determined by using one-way analysis of variance followed by Dunnett's test and SNK-q for multiple comparisons. The survival data among groups were analyzed by the *χ*^2^ test, and the survival rate was expressed with a Kaplan–Meier analysis. The statistical significance was accepted as *p* < 0.05.

## 3. Results

### 3.1. Survival Rate

**Figure 2:**
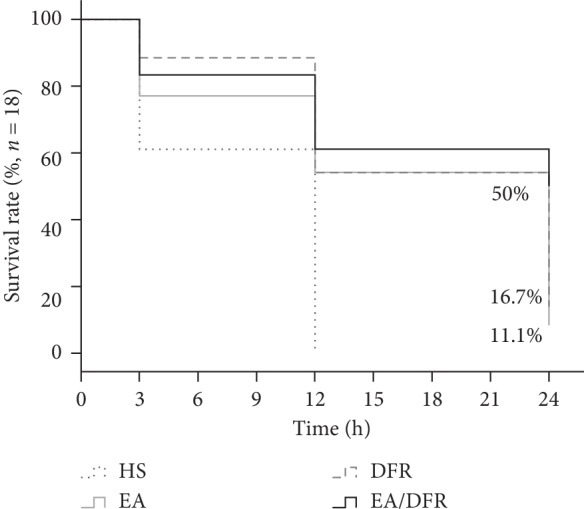
Kaplan–Meier graph of the survival rate per group over time. *n* refers to the original number of animals in each group. HS: hemorrhagic shock without fluid resuscitation; EA: electroacupuncture without fluid resuscitation after HS; DFR: HS with delayed fluid resuscitation; EA + DFR: electroacupuncture with delayed fluid resuscitation after HS.

The 24-survival rates of the HS group, EA group, DFR group, and EA + DFR group were 0%, 11.1%, 16.7%, and 50%, respectively, and the 24 h survival rate of the EA + DFR group was significantly higher than that of the DFR group (*t* = 2.51, *p*^a^ < 0.05) (see Figure 2).

### 3.2. Mean Arterial Blood Pressure (MAP)

**Figure 3:**
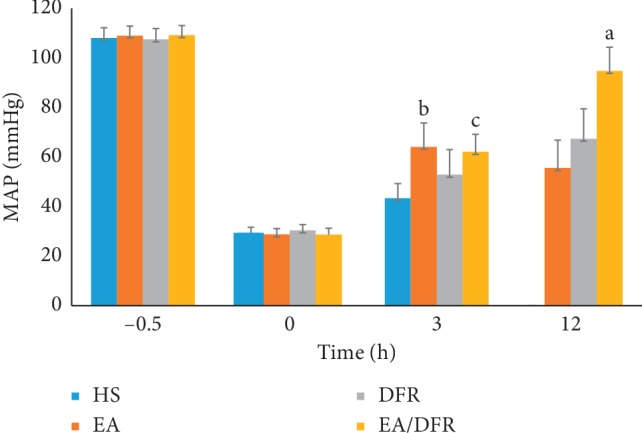
The change in the MAP per group over time. MAP: mean arterial pressure; HS: hemorrhagic shock without fluid resuscitation; EA: electroacupuncture without fluid resuscitation after HS; DFR: HS with delayed fluid resuscitation; EA + DFR: electroacupuncture with delayed fluid resuscitation after HS.

After the HS protocol, the MAP of each group dropped significantly (*p* < 0.01 each). The MAP of the EA/DFR group and EA group was significantly higher than that of the control group and DFR group at T3 (*p*^b^ < 0.05 and *p*^c^ < 0.05). At 12 h after hemorrhage, the level of the MAP in the EA + DFR group (94.77 ± 9.54 mmHg) was significantly higher than that in the DFR group (67.39 ± 12.03 mmHg) (*t* = 6.54, *p*^a^ < 0.01) (see Figure 3).

### 3.3. Visceral Blood Flow

**Figure 4:**
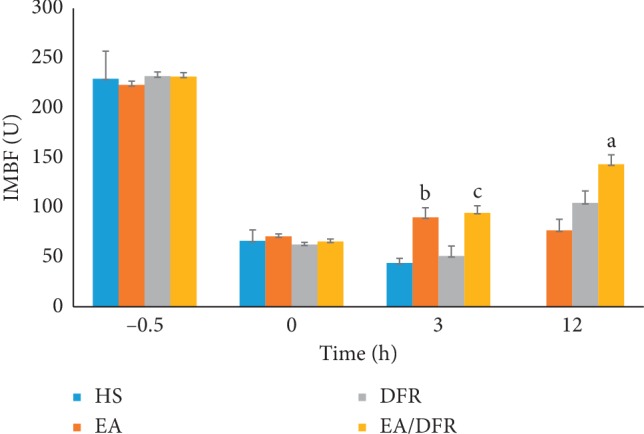
The change in the IMBF per group over time. IMBF: intestinal mucosal blood flow; HS: hemorrhagic shock without fluid resuscitation; EA: electroacupuncture without fluid resuscitation after HS; DFR: HS with delayed fluid resuscitation; EA + DFR, electroacupuncture with delayed fluid resuscitation after HS.

After the HS protocol, the intestinal mucosal blood flow (IMBF) of each group dropped significantly (*p*^b^ < 0.01 and *p*^c^ < 0.01). The IMBF of the EA + DFR group and EA group was significantly higher than that of the HS group and DFR group at T3 (*p* < 0.01 each). At T12, the IMBF of the EA + DFR group was significantly higher than that of the other groups (*p*^a^ < 0.01 each) (see Figure 4).

**Figure 5:**
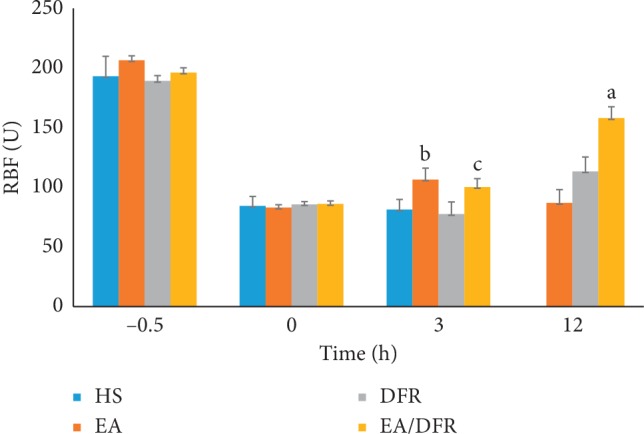
The change in the RBF per group over time. RBF: renal blood flow; HS: hemorrhagic shock without fluid resuscitation; EA: electroacupuncture without fluid resuscitation after HS; DFR: HS with delayed fluid resuscitation; EA + DFR: electroacupuncture with delayed fluid resuscitation after HS.

Similarly, the renal blood flow (RBF) of each group dropped significantly (*p* < 0.01 each) after the HS protocol. The RBF of the EA + DFR group and EA group was significantly higher than that of the HS group and DFR group at T3 (*p*^b^ < 0.01 and *p*^c^ < 0.01). At T12, the RBF of the EA + DFR group was significantly higher than that of the other groups (*p*^a^ < 0.01 each) (see Figure 5).

**Figure 6:**
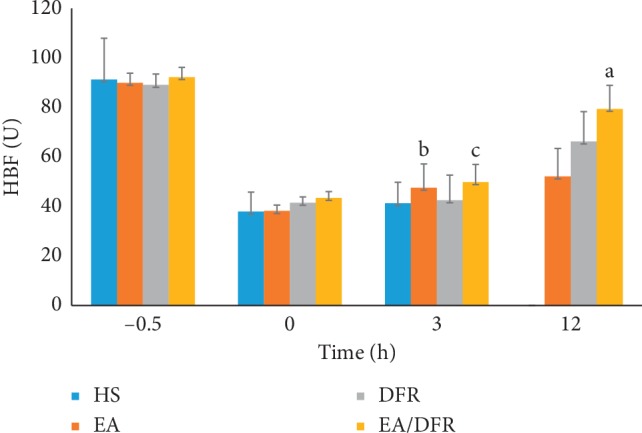
The change in the HBF per group over time. HBF: hepatic blood flow; HS: hemorrhagic shock without fluid resuscitation; EA: electroacupuncture without fluid resuscitation after HS; DFR: HS with delayed fluid resuscitation; EA + DFR: electroacupuncture with delayed fluid resuscitation after HS.

The hepatic blood flow (HBF) of each group also dropped significantly (*p* < 0.01 each) after the HS protocol, similarly to the IMBF and RBF. The HBF of the EA + DFR group and EA group was significantly higher than that of the HS group and DFR group at T3 (*p*^b^ < 0.01, *p*^c^ < 0.01). At T12, the HBF of the EA + DFR group was significantly higher than that of the other groups (*p*^a^ < 0.01 each) (see Figure 6).

### 3.4. Tissue Moisture Content

**Figure 7:**
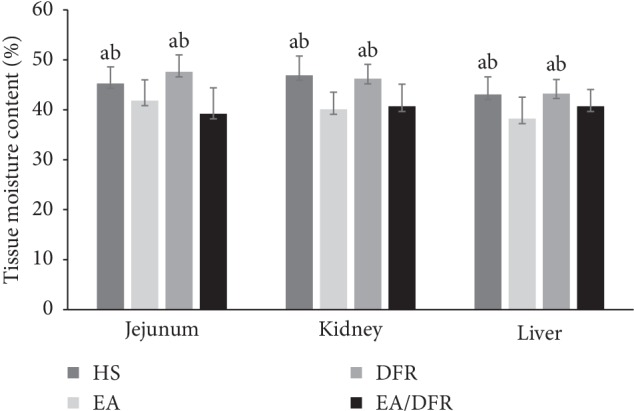
Measurements were carried out at T3. Note: the comparison with the EA group, ^a^*p* < 0.05; the comparison with the EA + DFR group, ^b^*p* < 0.05. HS: hemorrhagic shock without fluid resuscitation; EA: electroacupuncture without fluid resuscitation after HS; DFR: HS with delayed fluid resuscitation; EA + DFR: electroacupuncture with delayed fluid resuscitation after HS.

At T3, the moisture content of the jejunum, kidney, and liver of the groups receiving EA (EA group and EA + DFR group) was significantly lower than that of the groups not receiving EA (HS group and DFR group) (*p* < 0.01 each) (see Figure 7).

### 3.5. Blood Gas Analysis and Organ Function

Three hours after hemorrhage, the levels of lactate, PaCO_2_, ALT, and Cr of the groups receiving EA were significantly lower than those of the non-EA groups (*p* < 0.01 each), and the levels of pH, PaO_2_, and DAO of the groups receiving EA were significantly higher than those of the non-EA groups (*p* < 0.01 each) (see Table 1).

## 4. Discussion

Despite growing evidence of the experimental beneficial effects of EA on HS [9–11], there is scant evidence on the efficacy of EA for severe or fatal HS. Therefore, in an effort to approximate true austere environments, the effects of EA at ST36 on HS rats with 60% blood loss, including subsequent errhysis for 170 minutes, were investigated. The present results showed that the survival rate of the EA + DFR group was significantly higher than that of the DFR group and the other groups at T24 (*p* < 0.01), which indicated that EA at ST36 can improve the effects of delayed fluid resuscitation, postpone the development of HS, and finally raise the 24 h survival rate. However, it is worth mentioning that the abovementioned good effects can be achieved only by EA combined with fluid infusion, and EA alone did not improve the 24 h survival rate according to the result of the EA group. Moreover, the results showed that the MAP of the EA + DFR group was significantly higher than that of the other groups at T24, which was similar to the 24 h survival rate. As is apparent from Figures 3–5, EA at ST36 significantly increased the IMBF, RBF, and HBF compared with the non-EA groups at T3. Then, the same results were found for the tissue water content and organ function (Figure 7). The above results indicated that EA can maintain organ function by benignly regulating the MAP, visceral perfusion, and tissue oxygenation in the early stage of HS and buy time for the subsequent transfusion to reduce the death rates for HS animals with 60% blood loss, which is in accordance with the effects reported in the previous studies with 45% blood loss [12, 13].

To investigate how EA at ST36 protects organ function of HS, our previous studies found that abdominal vagotomy and *α*-bungarotoxin could weaken or eliminate the effects of EA at ST36 on rats with HS and indicated that the protective effect of EA at ST36 may be related to the cholinergic anti-inflammatory pathway stimulated by the vagus [13, 14]. Meanwhile, other studies have also proven that the protective effect of EA on HS can be interpreted to inhibit the release of TNF-*α* and IL-6 in plasma and reduce the inflammatory responses of HS, which is possibly related to an intact vagus nerve and cholinergic *α*7 nicotinic acetylcholine receptor [20–27]. As is known, the primary mechanism of HS in the early stage is hypoxic and ischemic injury caused by insufficient blood perfusion, which results in the irreversible development of shock, while inflammatory response is the secondary factor in the later stage. However, in this study, EA at ST36 played a role in maintaining the MAP and improving oxygen delivery within three hours after HS, so it is difficult to explain this good effect of EA on the early stage of HS with the cholinergic anti-inflammatory pathway.

In the early stage of HS, due to the quick decrease in blood volume, adrenal medullary hormone will be produced from the adrenal gland by activating the sympathetic adrenomedullary system to strengthen the systole, increase the peripheral vascular resistance, and redistribute the body blood flow [28]. The compensatory mechanism above is beneficial to maintaining relative stability of the circulatory system. However, there are some potential risks, and the primary one is that priority is given to the heart and brain's blood supply at the expense of the blood supply of other organs [29, 30]. For example, renal ischemia can lead to acute renal impairment, and gastrointestinal ischemia will damage the intestinal mucosal barrier, induce the migration of bacteria and toxins, generate endotoxemia, and ultimately lead to multiple organ failure. Therefore, if we are able to increase the excitability of the parasympathetic nervous system appropriately to inhibit excessive excitation or improve the imbalance of sympathetic and parasympathetic systems, the rapid development of HS will be delayed. Our preliminary research [31] found that EA at ST36 can increase the release of dopamine to improve the IMBF and ameliorate damage to the intestinal barrier by regulating the balance of sympathetic and parasympathetic systems. Hence, it is worth continuing to explore whether the good effects of EA in this study are related to the release of catecholamine and activation of the sympathetic and parasympathetic systems.

There were several limitations in this study. First, the measurement of organ function and the tissue moisture content cannot be carried out at several time points in an experiment due to the high mortality, which may produce additional errors. In addition, for extreme HS, this experiment mainly focused on the therapeutic effects of EA instead of further molecular biological mechanisms, and the roles of hormonal factors deserve more research. The third problem is the number of animals. If a larger number of subjects had been included, the results may have been more reliable.

## 5. Conclusion

In conclusion, the study findings suggest that the EA therapy followed by delayed fluid resuscitation can prolong the survival time of hemorrhagic animals with 60% blood loss and improve their organ function and tissue perfusion, which has the potential application value in harsh environments without intravenous fluids.

## Figures and Tables

**Table 1:** Selected laboratory values.

	pH	LA (mmol/L^−1^)	PaCO_2_ (mmHg)	PaO_2_ (mmHg)	ALT (U/L^−1^)	Cr (*μ*mmol/L^−1^)	DAO (U/L^−1^)
EA and EA + DFR	7.18 ± 0.09	7.19 ± 1.11	41.34 ± 3.45	70.96 ± 4.53	98.35 ± 10.37	73.5 ± 6.84	27.39 ± 2.94
DFR and HS	7.04 ± 0.06^a^	9.04 ± 1.26^a^	49.09 ± 3.09^a^	66.31 ± 4.30^a^	143.42 ± 15.58^a^	84.1 ± 7.30^a^	20.05 ± 2.53^a^

Measurements were carried out at T3. Note: the comparison with groups receiving EA, ^a^*p* < 0.01; HS: hemorrhagic shock without fluid resuscitation; EA: electroacupuncture without fluid resuscitation after HS; DFR: HS with delayed fluid resuscitation; EA + DFR, electroacupuncture with delayed fluid resuscitation after HS.

## Data Availability

The data used to support the findings of this study are available from the corresponding author in China upon request.
